# Mortars for Conservation of Late 19th and Early 20th Century Buildings—Combination of Natural Cements with Air Lime

**DOI:** 10.3390/ma15103704

**Published:** 2022-05-22

**Authors:** Slavka Andrejkovičová, Hamid Maljaee, Diana Rocha, Fernando Rocha, Maria R. Soares, Ana Velosa

**Affiliations:** 1 Geosciences Department, Geobiotec Research Unit, Campus Universitário de Santiago, University of Aveiro, 3810-193 Aveiro, Portugal; tavares.rocha@ua.pt; 2 Civil Engineering Department, RISCO, Campus Universitário de Santiago, University of Aveiro, 3810-193 Aveiro, Portugal; h.maljaee@ua.pt (H.M.); diana.vizinho.rocha@gmail.com (D.R.); avelosa@ua.pt (A.V.); 3 CICECO & LCA, Campus Universitário de Santiago, University of Aveiro, 3810-193 Aveiro, Portugal; rosarios@ua.pt

**Keywords:** 19th and 20th century architecture, natural cement, lime, mortars, characterization

## Abstract

With the availability of commercial Natural cements (NC) for the conservation purposes raises a fundamental question about the compatibility between historic and repair mortars. The properties of Natural cements are dependent on the geological location of the raw material extraction and also on the production parameters, both having an impact on the final properties of the mortars produced from each distinct. Therefore, the significance of preservation of 19th and 20th century heritage and selection of the proper binder compatible with the original materials necessitate the study of existing NCs, that nowadays are produced by several manufacturers. This work provides a complex study of the mortars prepared from three NCs available in the market: Groupe Prompt Vicat, France (NCPV); Cemento Collet Marfil (NCM) and Cemento Natural Tigre (NCT), both from Spain. Various mortar sets based on individual NC containing different binder/aggregate ratios and air lime additions were analyzed after 28, 60, and 90 days of curing with the focus on their mineralogical composition (XRD), morphology (SEM), mechanical (flexural and compressive strength), and physical properties such as water absorption by capillarity, water vapor permeability, and water vapor diffusion resistance. Mortars prepared from NCPV, NCM, and NCT show distinct physical-mechanical properties with varying binder/aggregate ratio and air lime addition. This study shows that the NC variability should be taken into consideration when selecting materials for the conservation and rehabilitation of historic renders and plasters. Based on the comparison with original NC mortars, several NC mortars developed in this study show adequate properties for conservation of the buildings from late 19th and early 20th century in terms of compressive strength (>12 MPa), water absorption by capillarity (<20 kg·m^−2^·h^−0.5^), water vapor permeability (<4 × 10^−10^ kg·s^−1^·m^−1^·Pa^−1^), and water vapor diffusion resistance (<28) values.

## 1. Introduction

Roman cement was patented by James Parker in 1796 [1], reflecting the burning of a septarian marlstone, followed by grinding, resulting in a brown material with rapid setting. Similar to the lime and hydraulic lime binders commonly used at the time, Roman cement, also known as Natural cement, was produced without additions to the raw materials, mainly composed of clay-rich marlstones, differing from the formers by requiring grinding instead of slaking before use. The convention adopted for this paper is that the term “Natural cement” is applied to Roman cements. The original marl limestones should have a clay content between 22 and 35% resulting in a primarily hydraulic set due to the high content of clay and low free lime [2,3,4]. The final properties of Natural cements are directly influenced by the geological location of the sourced marlstone. This results in distinct characteristics such as color and chemical composition.

The mineralogy of the Natural cements is a result of the raw materials composition and the manufacturing process depends on the time and temperature of calcination. Compared to Portland cement, the burning temperature is lower, between 850 and 1100 °C, resulting in the production of belite as the main hydraulic phase. Simultaneously, usually, there is the formation of calcium aluminates that will hydrate and result in calcium aluminum hydrates, giving the binder its rapid set [2,5,6]. The study of the production of modern Natural cements concluded that ideal cements with high strength and rapid setting resulted from low temperature manufacturing. The main crystalline phase in these cements is belite: α-belite for lower production temperatures changing into β-belite for higher manufacturing temperatures [7].

By the end of the 18th century and the beginning of the 19th century, Natural cement was mainly produced in England [8]. In 1802, France started to industrially produce Natural cement with several production locations starting their manufacturing from 1840. At the same time, after importing Natural cement from England and the first local production in 1820, Germany also began to produce it on a large scale. Austria, one of the countries where Natural cement was largely produced, started its major production in 1842 [9]. With the development of Portland cement (OPC), the production of Natural cement declined, primarily affecting English production and later the rest of Europe, with only a few manufacturers maintaining this binder in production. Nowadays some of the best-known producers are: Groupe Vicat, France; Cementos Collet Marfil and Cemento Natural Tigre, both from Spain [10]. Natural cement can be found in many decorative elements of the building facades from the 19th century to the early 20th century. It was used due to its relatively rapid setting and a proper ability to be manipulated for the production of exterior ornaments showing a good resistance to the weather. A distinctive feature of most historic Natural cement mortars is their high porosity accessible to water (30–40% by volume), combined with generally high mechanical strength and excellent durability [8]. The binder can be found all across Europe and was used in numerous buildings in Austria and Poland [8,11]. Recent studies suggest the presence of Natural cement in Portuguese heritage buildings particularly around the second half of the 19th century and the beginning of the 20th century [12,13], thus showing the relevance of this study. With the availability of Natural cements for the conservation purposes, important questions about the compatibility between historic and repair mortars must be answered, as the compatibility is broadly defined as the capacity of the repair mortar to interact with the original historic material without causing any damage [14,15,16]. The main requirements for repair mortars include limited water absorption, high water vapor permeability, high flexibility, good adhesion, durability, and compatibility with the substrate [17]. Therefore, the selection of materials compatible with historical structures is therefore very important and needs a complex solution. Research into Natural cements as materials used in the monumental restoration is a novel subject, because the use of such materials has been recently rediscovered and their historicity has been taken into account.

Therefore, the main objective of the present paper is the development of modern binders compatible with the existing materials in heritage buildings from the end of the 19th and the beginning of the 20th century. To accomplish this objective, this study has three aims: (a) chemical and mineralogical characteristics of three Natural cements available in the market such as Groupe Prompt Vicat (France), Cementos Collet Marfil and Cemento Natural Tigre (both from Spain) and their comparison with those of air lime and a natural hydraulic lime, the binders common for restoration purposes; (b) analysis of mortars with different binder/aggregate ratios and lime additions to verify their influence on the final mortars’ characteristics; and (c) a complex study of the mineralogical composition, morphological, mechanical, and water absorption properties of the mortars from the view of their use as repair mortars for buildings from the late 19th and early 20th century.

## 2. Materials and Methods

### 2.1. Raw Materials and Mortars

**Figure 1:**
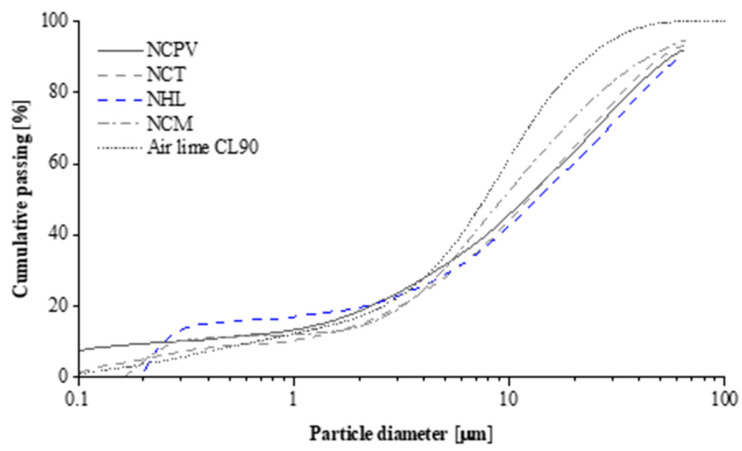
Particle size distribution of the binders.

Three binders were selected for the development of Natural cement (NC) mortars: *Prompt Vicat* (“Ciment Naturel Prompt Vicat” NCPV; Groupe Vicat, Grenoble, France), *Marfil* (“Cemento Natural Marfil” NCM; Cementos Collet, Barcelona, Spain), and *Tigre* (“Cemento Natural Rápido Tigre” NCT; Cemento Natural Tigre, Barcelona, Spain). Moreover, two lime-based binders: air lime CL90 (H100 Lusical, Alcanede, Portugal) and natural hydraulic lime (NHL 3.5; SECIL, Lisbon, Portugal) were chosen for comparison. The particle size distribution of individual binders is illustrated in Figure 1. NCs show a similar distribution trend, while air lime and NHL 3.5 display a slightly different particle size distribution. It can be seen that about 70% of air lime particles (D_50_ = 7.69 µm) are finer than NHL 3.5 and Natural cements. NHL 3.5 contains the largest grains among all the binders with D_50_ = 16.28 µm, while NCT, NCVP, and NCM show D_50_ values of 14.45, 14.32, and 10.88 µm, respectively. The aggregate used was siliceous river sand (D_50_ = 0.3 mm).

The volumetric formulations and their designations are represented in Table 1. Mortars were formulated with various binder/aggregate (b/a) ratios aiming to produce the type of mortars commonly used for conservation and repair of buildings of this period, with the emphasis on the fact that the mixture must be much richer in cement than usual mortars for rendering [8]. Air lime was used to replace a part of the hydraulic binders in some formulations to explore the resulting characteristics, as it is known that the original Natural cement mortars often contained lime [18]. 

### 2.2. Methods

#### 2.2.1. Raw Materials

The conversion of volumetric formulations to mass quantities was calculated using the specific gravity of the materials calculated following the EN 1015-6.1998 [19] procedure.

The particle size distribution of fine materials was determined with an X-ray grain size analyzer Sedigraph III Plus from Micromeritics (Barcelona., Spain) using Sodium Hexametaphosphate (1%) as a dispersant. 

Mineralogical composition of randomly oriented powdered samples was performed by the X’Pert-Pro M.P.D. Philips/PANalytical X-ray diffractometer (Almelo, Netherlands). The operating conditions were 30 mA and 50 kV. The scan was performed between 4° and 60° 2θ by using the CuKα radiation (λ = 1.5405 Å) at a speed of 0.02°/s. The XRD phase detection was performed by Highscore Plus 4.9 with ICDD database PDF4+ (2021).

Chemical analysis of the major and minor elements was carried out by wavelength dispersive PANalytical PW 4400/40 Axios X-ray fluorescence spectrometer (Almelo, Netherlands) with CrKα radiation. A pressed disc (≈115 MPa) with about 4 cm diameter and 0.5 cm height was previously prepared with 10 g of powder and 5 drops of polyvinyl alcohol. Loss on ignition was determined by gravimetric analysis, by calcinating the sample at 1000 °C for 1 h. The experimental error is within 1% relative.

#### 2.2.2. Mortars

##### Mortars Production, Curing Conditions, and Wet State Tests

Mortar preparation started by weighing and mixing the dry materials for homogenization and then adding water following the procedure of the EN 1015-2:1999 [20] The amount of added water was determined by trial mixes to achieve the desired workability measured by the flow table test following the EN 1015-3:1999 [21] Standard. 

Standard steel molds were used to prepare the mortar prisms with the dimensions 160 × 40 × 40 mm and PVC molds for the disc-shaped samples with 130 mm diameter and 20 mm thickness. Samples were conditioned at 20 ± 2 °C and 95 ± 5% RH for the first 7 days and at 20 ± 2 °C and 65 ± 5% RH for the rest of the curing time, following the EN 1015-2:1999 [20]. After removing from the molds, specimens were maintained at a relative humidity of 65 ± 5% and cured up to 28, 60, and 90 days.

Mortar’s formulations and water amount (wt.% of the total solid mass) added during mortars preparation are presented in Table 1.

##### Dry State Testing

Mineralogical composition of randomly oriented powdered samples was performed by the X’Pert-Pro M.P.D. Philips/PANalytical X-ray diffractometer. The operating conditions were 30 mA and 50 kV. The scan was performed between 4° and 60° 2θ by using the CuKα radiation (λ = 1.5405 Å) at a speed of 0.02°/s. The XRD phase detection was performed by Highscore Plus 4.9 with ICDD database PDF4+ (2021).

Flexural and compressive strength tests were carried out on mortar prisms (160 × 40 × 40 mm) on a Precision Universal Tester equipment (SHIMADZU: AG-IC 100 kN, Tokyo, Japan), by applying a maximum force of 100 kN at the speed of 50 N/s according to the Standard EN 1015-11 [22].

For the determination of the depth of carbonation, after the flexural strength test, the exposed inside of the samples was sprayed with a 0.2% phenolphthalein solution, after which the depth of the uncolored area, the carbonation front, was measured in a direction perpendicular to the side of the sample. 

The water absorption by capillarity was determined following the procedure in the EN 1015-18:2002 [23] Standard at 28, 60, and 90 days of curing. Two mortars specimens obtained after the flexural strength test were analyzed in parallel and the final value of the certain sample is reported as an average of two measurements. 

The water vapor permeability of the disc-shaped mortars was determined according to the EN 1015-19:1999 [24] Standard using the *wet cup* method. The water vapor resistance factor (*μ*) was measured according to the following equation:

*μ = δ_A_/WVP* (1)

where *δ_A_* is air permeability (1.94 × 10^−10^ kg/(Pa m s)) in test conditions (20 °C and 50% RH) and *WVP* is water vapor permeability.

The microstructural characterization was carried out by scanning electron microscopy (SEM–Hitachi, SU 70, Tokyo, Japan) and energy dispersive X-ray spectrometry (EDS–EDAX with detector Bruker Quantax 400 AXS (Bonsai Advanced, Madrid, Spain), operated at 3–30 kV. Before the scanning, all the samples were coated with carbon to enhance the electric conductivity.

## 3. Results and Discussion

### 3.1. Characterization of Materials Used for Mortar Preparation

Binders used in this work present distinct visual characteristics. Air lime and NHL showed lighter color, white, and light grey, respectively, while the three Natural cements’ colors were within the brown gamut.

#### 3.1.1. Specific Gravity

Determination of the binder and aggregate specific gravity was mainly used for the calculation of the masses for the individual cement formulations. In addition, it allowed to determine differentiating characteristics within the binders, particularly between the Natural cements (Table 2).

In the case of binders, the lowest value of specific gravity shows air lime (385 kg/m^3^). NHL and the NC’ specific gravities present intermediate values (744–911 kg/m^3^) with some noticeable variables between the NC binders. The specific gravity of siliceous river sand is uppermost (1559 kg/m^3^) among all the materials.

#### 3.1.2. X-ray Fluorescence Analysis (XRF)

Chemical analysis obtained by XRF (Table 3) summarizes the major elements present in materials used for mortars preparation. As expected, all the binders used in this study show a high percentage of CaO, ranging from 50.5 (NCPV) to 65.6 wt.% (NHL). At the same time, the next more prevalent oxides are SiO_2_ (~12.3–19 wt.%) and Al_2_O_3_ (5.3–7.8 wt.%). Hydraulic binders including NHL and three natural cements show a chemical composition as expected from the hydraulic nature of these binders. The presence of SiO_2_ yields the hydraulic capacity of the binder through the formation of polymorphs of belite (C_2_S). Natural cements are characterized by a higher content of silica (the maximum occurs in NCM with 19 wt.%) than NHL. However, air lime, which is produced through the calcination of limestone (CaCO_3_), lacks this reactivity due to the absence of silica, as expected.

All types of natural cements are characterized by significant quantities of SO_3_ ranging between 2.16 and 4.12 wt.%, which are common values in commercial NCs due to the presence of sulfates in the raw materials [10,25]. Sulphate presence may promote the formation of ettringite in presence of aluminum and calcium during the hydration process (this will be discussed in Section 3.2.1). Fe_2_O_3_ ranging between 2.32 and 2.97 wt.% is close to the values of original NC mortars (2.13 wt.%) studied by [26]. 

NCs are also found with a higher K_2_O content (1.25–1.50 wt.%) compared to NHL, which is associated with the presence of minerals such as feldspar and mica in the raw cement materials. As expected, NCs contain higher alumina content (5.26–7.83 wt.%) compared to NHL (1.66 wt.%). Al_2_O_3_ in natural cements is responsible for their quick setting. NCT and NCPV show similar values of 5.3 and 5.9 (wt.%), respectively; while the content of Al_2_O_3_ in NCM is higher (7.8 wt.%). MgO content, which may be due to the presence of dolomitic aggregates, varies significantly within NCs between 1.5 (NCM) and 6.0 (NCT) wt.%. Chemical analyses of Natural cements in this study are in a good agreement with those of original Natural cement studied by [27]. The sand used in this work is mostly siliceous (96.5 wt.%) with minor quantities of Al_2_O_3_ and K_2_O (Table 3).

#### 3.1.3. X-ray Diffraction Analysis (XRD)

Mineralogical composition of the materials is shown in Table 4. The main crystalline phases in air lime (CL90) and natural hydraulic lime (NHL 3.5) are portlandite and calcite. Furthermore, NHL 3.5 shows the presence of belite (*β*-C_2_S) reflecting the hydraulic character of this binder. 

All three Natural cements still contain an undecomposed calcite and dolomite, together with typical products of calcination such as belite, which is present in two polymorphs: *α*′-C_2_S and *β*-C_2_S. Additionally, high temperature phases (>1100 °C) such as gehlenite (C_2_AS) and brownmillerite (4CaO·Al_2_O_3_·Fe_2_O_3_) were as well identified in all NCs. C_2_S carbonation during calcination is reflected in the production of both spurrite-Ca_5_(SiO_4_)_2_(CO_3_) and tilleyite-Ca_5_Si_2_O_7_(CO_3_)_2_, in accordance with the study of Hughes et al. (2019). Additionally, all cements display evidence of incomplete calcination as demonstrated by the presence of residual quartz. Refs. [10,28] also detected anhydrite, periclase, and ye’elimite in these natural cements, but these phases were not identified by XRD in the present study.

### 3.2. Mortar Characteristics

#### 3.2.1. X-Ray Diffraction Analysis (XRD)

**Figure 2:**
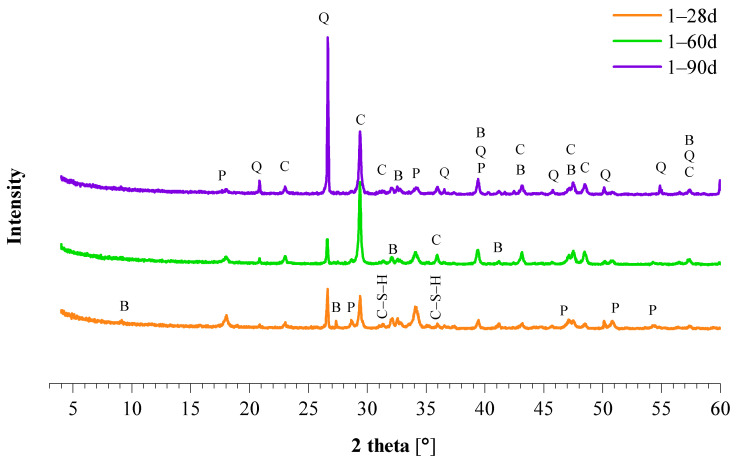
XRD analysis of 1_NHL mortar. B—belite, C—calcite, C–S–H—calcium silicate hydrate, P—Portlandite, Q—quartz.

The study of the development of the new phases during hardening of the mortars was performed using XRD analysis. Mineralogical composition of representative mortars 1_NHL 3.5, 3_NCPV, 10_NCM, and 17_NCT (from Table 1) after 28 days of curing is summarized in Table 5. Hardening of hydraulic limes is a combination of hydration, by the formation of a calcium-silicate-hydrate (C–S–H) and carbonation through the reaction between the portlandite and CO_2_ from the atmosphere [29]. Presence of calcite and formation of C–S–H due to C_2_S hydration was confirmed in 1_NHL 3.5 mortar (Table 5), although calcite was already present in the mineral matrix of NHL 3.5. Unreacted portlandite, belite, and quartz were also identified in 1_NHL 3.5 mortar. Mineralogical composition of 1_NHL 3.5 mortar after 28, 60, and 90 days of curing is shown in Figure 2. No obvious changes in mineralogy were observed during mortar hardening, apart from the decreasing amount of portlandite with curing time due to its transformation to calcite, however visible portlandite peaks are still observed after 90 days of curing. The presence of portlandite at later ages can also be the result of the belite hydration.

**Figure 3:**
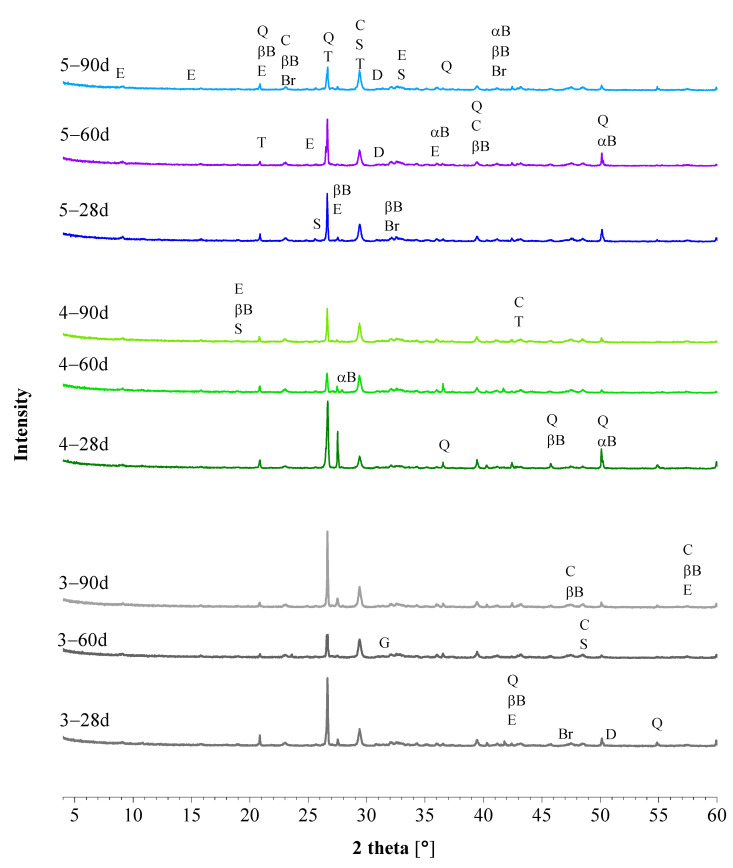
XRD analysis of NCPV mortars: 3_NCPV, 4_NCPV and 5_NCPV (b/a ratio 1:0.5, 1:1 and 1:2, respectively). αB—alpha belite, βB—beta belite, Br—brownmillerite, C—calcite, D—dolomite, E—ettringite, G—gehlenite, Q—quartz, S—spurrite, T—tilleyite.

The XRD patterns of natural cement mortars contain all reflections of crystalline phases present in the original cements (Table 5). Another similarity between NC mortars is that the only hydration phase formed is ettringite. The presence of ettringite is linked to the sulfates present in the binders [26]. Although anhydrite or gypsum were not identified in natural cements by XRD, this does not rule out their presence as an amorphous phase or below the detection limit of the XRD instrument. Moreover, chemical analysis of NCs (Table 3) shows relatively high sulfur content reflecting the presence of sulfates in the raw materials. Figure 3 depicts the mineralogy of the representative mortars produced from NCPV, after 28, 60, and 90 days of curing: 3_NCPV, 4_NCPV, and 5_NCPV with the binder/aggregate ratio of 1:0.5, 1:1, and 1:2, respectively. As in the case of NHL mortar (Figure 2), no significant changes in the mineralogy are observed with increased curing time. Brownmillerite, present in all NC mortars, remains inert during hydration as its reflections are noticeable even after 90 days of curing. Mineralogical characteristics of NCM and NCT mortars were analogous to the ones obtained from the XRD analysis of NCPV mortars, thus for this reason the figures are not included in the text. Furthermore, NC binders combined with CL90 resulted in mortars with identical phase composition apart from the detection of portlandite mainly in early ages of curing and more noticeable for the formulation richer in lime, and for this reason they are not shown in the text.

#### 3.2.2. SEM-EDS Analysis

**Figure 4:**
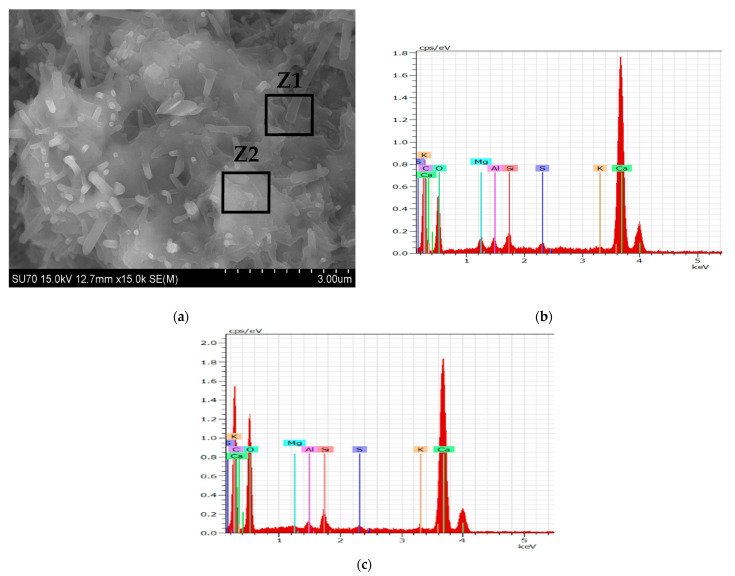
SEM micrograph of 1_NHL mortar. (**a**) with EDS spectra for Z1 (**b**), and Z2 (**c**).

**Figure 5:**
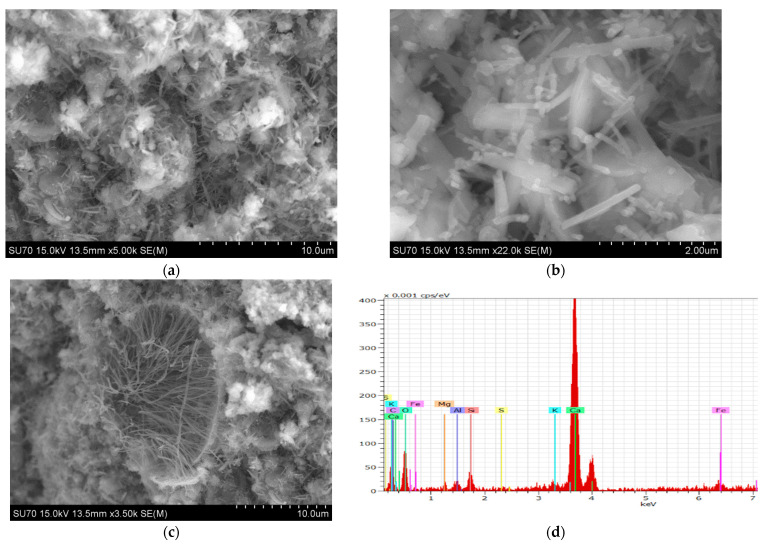
(**a**–**c**) SEM micrograph of 3_NCPV, (**d**) EDS spectrum of the needle-shaped particles.

**Figure 6:**
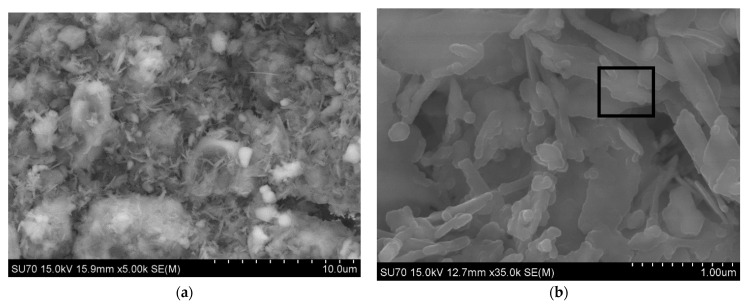

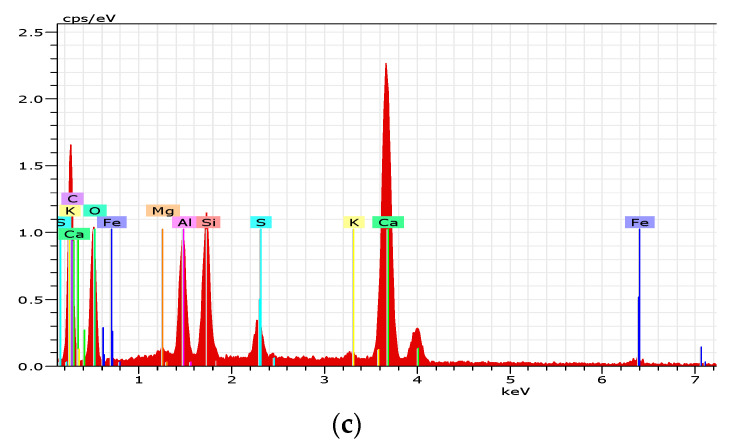
(**a**,**b**) SEM micrograph for 10_NCM mortar, (**c**) EDS spectrum from zone identified in (**b**).

**Figure 7:**
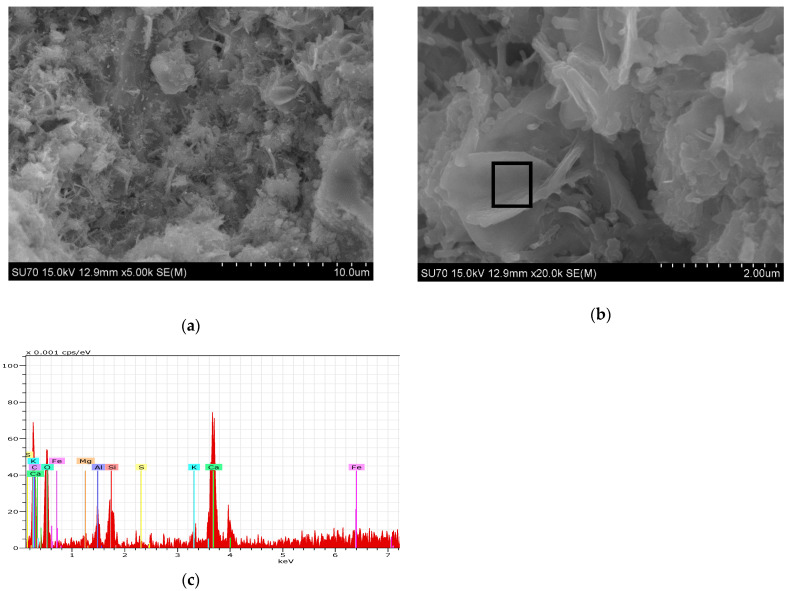
(**a**,**b**) 17_NCT mortar SEM micrographs, (**c**) EDS spectrum from zone identified in (**b**).

For the generality of the mortars, the data from SEM and EDS analysis of the studied samples cured for 28 days show the microstructure expected from the hydraulic binders. 1_NHL mortar (Figure 4) demonstrates a dense matrix of C_2_S with the needle-shaped morphologies related to C–S–H precipitation (both detected in XRD analyses, Figure 2, Table 5). The microstructure of the NCPV mortar manufactured with 1:0.5 binder/aggregate ratio is characterized by closely packed hydration products combining floccular and needle-shaped morphologies (Figure 5). The detail on ettringite needles filling the pore illustrates Figure 5c. NCM mortar with the same binder/aggregate volumetric ratio presents a similar microstructure (Figure 6) and detected elements as NCPV. Particles with Ca, Al, and S indicate the formation of ettringite (Figure 6b,c). NCT mortar is characterized by a compact microstructure with an assemblage of ettringite needles along with the NCT matrix (Figure 7a). More compact structure of NCT mortar is probably related to the size of NCT particles, which are finer compared to NCM and NCT (Figure 1).

#### 3.2.3. Flexural and Compressive Strength

**Figure 8:**
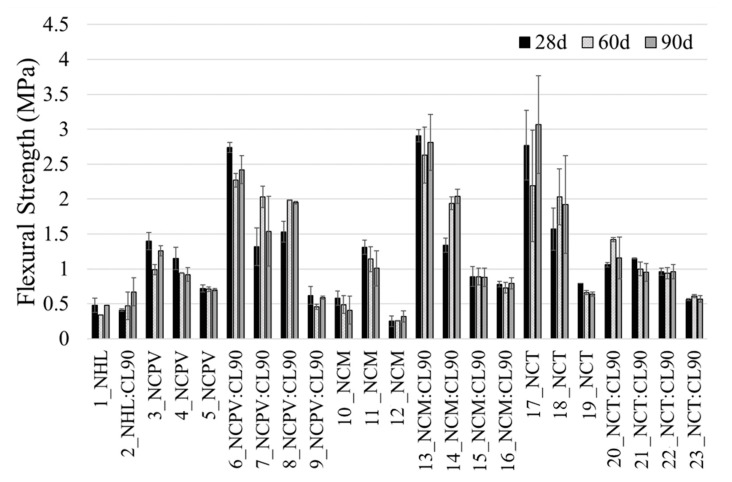
Flexural strength of the mortars.

**Figure 9:**
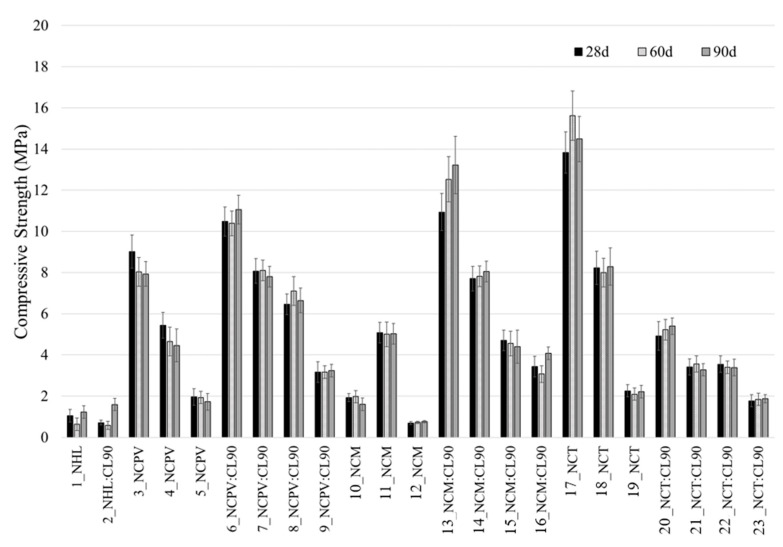
Compressive strength of the mortars.

Figure 8 and Figure 9 present the average flexural (*Rf*) and compressive strength (*Rc*) values of the mortars analyzed after 28, 60, and 90 days of curing. No gradual increase of mechanical properties is observed with curing time, on the contrary, majority of the mortars show comparable values of the strengths at 28, 60, and 90 days. This phenomenon is associated with the characteristic of NC mortars, which is rapid hardening, that reflects in high initial and long-term strength (e.g., [10]). However, many mortars exhibit a drop in flexural and compressive strength from 28 to 60 days, or an increase from 28 to 60 days followed by decrease of the values at 90 days of curing. This might be related to the hydration mechanism of NC mortars. The decrease of compressive strength in NC paste from 21 MPa (4 months of curing) to 18 MPa (6 months of curing) was also observed in [30] and little or increase in *Rc* from 28 to 90 days of the mortars containing NC:lime:sand (0.5:0.5:1.5) observed [18]. The long-term strengths in Natural cements are correlated with the formation of C–S–H gel, which is a result of belite hydration (Gosselin, 2013). Although C–S–H gel was not unambiguously detected in NC mortars by XRD and SEM analyses, as it was in the case of NHL mortars (Figure 2 and Figure 4), its presence cannot be ruled out as C–S–H gel is usually poorly crystallized or is present below the detection limit of XRD instrument. According to [30], the formation of the C–S–H gel correlates with the change in paste porosities, furthermore, the process is preceded by dormant periods of varying duration. Thus, the changes in porosity due to C–S–H formation could be a reason of differences between the values of mechanical properties within curing ages. Moreover, complete reaction of the original components has not occurred, as all NC mortars analyzed in this study show the presence of both polymorphs of unreacted belite during all ages of curing (Table 5, Figure 3), thus the possible hydration of belite above 90 days of curing should not be excluded.

Flexural and compressive strengths of individual mortars show similar trend (Figure 8 and Figure 9). Comparing the data at 90 days of curing, the lowest values of *Rf* and *Rc* shows 12_NCM (1:2 b/a) mortar (0.32 and 0.77 MPa, respectively), followed by 1_NHL and 2_ NHL: CL90 mortars (0.48 and 1.23 MPa, 0.67 and 1.60 MPa, respectively). Addition of CL90 to NHL mortar (2_NHL:90CL) improved flexural and compressive strengths values by 30 and 23%, respectively, when compared to NHL. 

At 90 days of curing, top values of *Rf* (~3 MPa) and *Rc* (~14.5 MPa) show 17_NCT (with 1:0.5 b/a ratio), followed by 13_NCM:CL90 (0.75:0.25:1 b/a) and 6_NCPV:90CL (0.75:0.25:1 b/a) with the flexural and compressive strength values of 2.75 and 13 MPa, and 2.4 and 11 MPa, respectively. Following these observations, apparently, each type of natural cement requires different formulation for mortar fabrication, to reach high mechanical strengths. Moreover, up to date, there is a lack of scientific papers related to mechanical resistances of the historic structures containing Natural cements; however, for example, according to [27], compressive strength of Natural cement mortar samples collected from Bourges Cathedral (France) varies between 12 and17 MPa. However, in a study carried out by [4], the compressive strengths of historic natural cement renders from 19th century reached 18.4 and 10.7 MPa, 18.4 MPa and 56.8 MPa for those blended with lime, rich in aggregates and poor in aggregates, respectively. 

Thus, the highest compressive strengths achieved by mortars 17_NCT, 13_NCM:CL90 and 6_NCPV:90CL, are compatible in terms of strength with those used in the original historic buildings. 

Binder/aggregate ratio plays an important role in the final values of mechanical properties of the mortars. Comparing individual NC mortar sets at 90 days, without CL90 addition, NCPV (samples 3, 4 and 5) and NCT (17, 18 and 19) display descending trend in *Rf* and *Rc* with higher aggregate proportion in b/a ratio in mortar formulation (1:0.5 > 1:1 > 1:2). NCM mortar shows an unusual behavior in which mechanical properties follow the trend: 1:0.5 < 1:1 > 1:2 b/a ratio (sample 11). Unexpectedly low mechanical performance of 10_NCM (1:0.5 b/a) in both *Rf* (0.41 MPa) and *Rc* (1.61 MPa) was observed, although this sample did not show any sign of pathologies or presence of microcracks. 

Lime (CL90) addition to the mortars had also impact on the mechanical properties. In the case of NCPV (sample 3_NCPV, 1:0.5 b/a) at 90 days, there is observed a rise in *Rf* of about 90% and about 40% in *Rc*, when mortar’s formulation is 0.75:0.25:1 (NC:CL90:sand) ratio (sample 6_NCPV:CL90). *Rc* in this sample thus reached ~11 MPa, which is a closer value to compressive strengths of Natural cements in the historic monuments [27]. Presence of lime in these mortars improved mechanical strengths, as subsequent portlandite carbonation, compared to rapid hydraulic reactions in NCs, is responsible for a strength gain. Other combinations in mortar’s formulation such as 0.5:0.5:1 (7_NCPV:CL90) and 0.75:0.25:1.5 (8_NCPV:CL90) also caused higher values of *Rf* (22 and 55%, respectively) compared to 3_NCPV, but on the other hand, did not improve compressive strength ones. 

In the case of NCM sets (samples 10_ NCM, 11_NCM and 12_ NCM), the highest mechanical resistances were obtained for 11_NCM (1:1 b/a) with *Rf* and *Rc* = 1 and 5 MPa, respectively and not for 1:0.5 (b/a) as in the case of NCPV (sample 3_NCPV) and NCT (sample 17_NCT). When lime was added to NCM to produce 0.75:0.25:1 (13_NCM:CL90) mortar, a vast increase in both *Rf* (about 180%) and *Rc* (about 160%) strength was observed, compared to 11_NCM. *Rf* and *Rc* of 14_NCM:CL90 (0.5:0.5:1) mortar are about 100 and 60% higher in comparison with 11_NCM, however these values are lower compared to 0.75:0.25:1 as it is in the case of NCPV mortar. Mortar’s formulation 0.5:0.5:1.5 in both NCPV and NCM does not cause any improvement in mortar’s mechanical resistances due to an excess of lime and high proportion of aggregate in the mixture, thus resulting in mortars with low mechanical performances. Low *Rc* values (~3 MPa) at 90 days of the same mortar formulation (0.5:0.5:1.5), although using different NC was observed by [18]. This means that optimum NCPV and NCM mortar’s formulation is 0.75:0.25:1 to reach high mechanical resistances. On the other hand, NCT mortar with 1:0.5 ratio (17_NCT) displays the highest *Rf* and *Rc* of all the mortars studied. Due to a dense and compact microstructure of 17_NCT, as confirmed by SEM analysis (Figure 7), less pore space for calcite formation is probably available and therefore lime addition in all formulations (20_NCT:CL90 to 23_ NCT:CL90) caused a drop in the final mechanical properties. 

#### 3.2.4. Carbonation Depth

The measurements of the carbonation front are presented in Table 6. The absence of color indicates that the portlandite was converted to calcite by carbonation or a pH below 8.2. Portlandite can appear due to its presence in the raw materials, mainly the NHL and CL90, or as a hydration product of the cement. NC Prompt Vicat and Marfil combined with CL90 showed a delayed carbonation front compared to the Natural cement mortars without lime in their formulation, which are fully carbonated after 28 (10_NCPM–12_NCM) and 60 (3_NCPV to 5_NCPV) days of curing. That means that the mortars with lime in their composition still contain unreacted material responsible for subsequent strength gain. This is in accordance with higher performances of mechanical properties of lime-combined mortars compared to those without lime in the formulation (Figure 8 and Figure 9).

NCT mortars without lime (17_NCT to 19_NCT) display a distinct behavior in terms of carbonation similarly as it is in the case of mechanical strengths (Table 6, Figure 8 and Figure 9). More compact structure resulting in the consequent refinement of the pores complicate the ingress of CO_2_, which reflects in the fact that these mortars are not fully carbonated after 90 days of curing.

#### 3.2.5. Water Absorption by Capillarity

**Figure 10:**
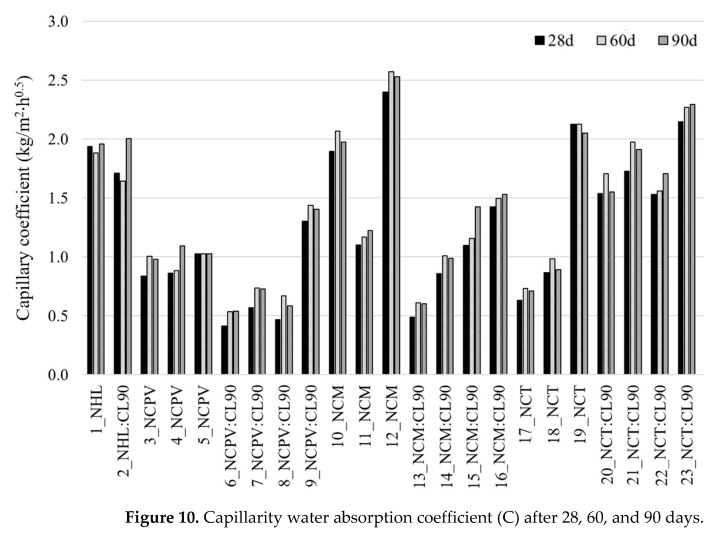
Capillarity water absorption coefficient (C) after 28, 60, and 90 days.

The degradation of historic buildings caused by excessive moisture resulting in the walls damages and failures is a common problem in the construction industry [31]. Thus, the low values of water adsorption by capillarity by repair mortars are essential. To evaluate the behavior of the mortars in contact with liquid water, the samples were subjected to the water absorption by capillary test after 28, 60, and 90 days of curing. The coefficients (*C*) resulting from this test are presented in Figure 10. Similarly, as in the case of mechanical properties (Figure 8 and Figure 9), no definite evolution of *C* is observed with increased time of curing, although the majority of the mortars show the lowest *C* at 28 days of curing. On the other hand, [10] analyzed mortars prepared from Natural cements Tigre, Marfil, and Prompt (1:3 b/a) and their *C* data show a drop from 28 (4.04, 2.81 and 5.50 kg·m^−2^·h^−0.5^, respectively) to 90 days of curing (2.29, 1.97 and 1.28 kg·m^−2^·h^−0.5^, respectively).

Comparing the mechanical performances of the mortars at 90 days (Figure 8 and Figure 9) with *C* (Figure 10), it is evident that the values of water absorption by capillarity are reverse to those of *Rf* and *Rc*. Mortars with low mechanical strengths show high *C* values and vice versa. This behavior was also observed in NC mortars studied by [10]. At 90 days, the lowest *C* values from individual NCPV, NCM, and NCT mortars’ sets show 6_NCPV:CL90 (4.07), 13_NCM:CL90 (4.65), and 17_NCT (5.50 kg·m^−2^·h^−0.5^), respectively. These are the mortars with the highest mechanical strengths (Figure 8 and Figure 9). Natural cement mortars collected from Bourges Cathedral (France) demonstrate the values of water absorption by capillarity between 8.6 and 9.3 kg·m^−2^·h^−0.5^ [27]. In the historic renders collected from 19th century European buildings by [4], the capillary coefficients varied from 4.42 to 12.75 kg·m^−2^·h^−0.5^ in the mortars rich in aggregates and poor in aggregates, respectively, and 22.67 kg·m^−2^·h^−0.5^ was *C* value obtained for Natural cement-lime blend. The repair mortars need to present compatible characteristics with remaining renders, thus the water absorption by capillarity values should not be higher than those in original mortars. All the mortars (Figure 10) show required values (<20 kg·m^−2^·h^−0.5^).

#### 3.2.6. Water Vapor Permeability and Water Vapor Diffusion Resistance Factor

Table 7 presents the water vapor permeability (*WVP*) and the water vapor diffusion resistance factor (*μ*) of the mortars at 28 days of curing. The main requirements for repair mortars include limited water absorption and high water vapor permeability. NHL mortars are the most permeable presenting *WVP* values ~1.61 × 10^−11^ kg·s^−1^·m^−1^·Pa^−1^, NCPV and NCM mortars show intermediate values between 1.23–1.35 × 10^−11^ kg·s^−1^·m^−1^·Pa^−1^ and NCT mortars are the least permeable with *WVP* coefficient between 0.60 and 0.70 × 10^−11^ kg·s^−1^·m^−1^·Pa^−1^. *WVP* values are strongly related to the open porosity of the mortars, thus NCT ones due to their compact structure are the least permeable of all the mortars. Nevertheless, the historic Natural cement mortars show an average *WVP* value ~4 × 10^−10^ kg·s^−1^·m^−1^·Pa^−1^ [8], therefore all the mortars analyzed in this study, besides NCT ones, show better *WVP* performances. Within the same binders, the different formulations have little to no impact on the *WVP* values.

Related to the water vapor diffusion resistance, NHL mortars present the least resistance to the vapor diffusion with *µ* = 11.85 (1_NHL) and 12.01 (2_NHL:CL90), NCM and NCPV mortars show intermediate values between 14.12 and 16.73 and the highest water vapor resistance was registered in NCT mortars with factors ranging between *µ* = 27.78 and 30.11. Compared to the study of [32], in which the authors analyzed vapor diffusion resistances of lime and OPC mortars, lower *µ* values of NHL mortars (11.85 and 12.01, samples 1 and 2, Table 7) are closer to those of lime mortars (*µ =* 11.09) and *µ* values over 20 are typical for OPC, which were observed for NCT mortars (Table 7). Moreover, in the study carried out by [4], historic Natural cement renders from 19th century present *µ* values 18, 20, and 28 in those blended with lime, poor in aggregates and rich in aggregates, respectively. As in the case of *WVP*, all the mortars besides NCT ones (*µ* > 28) reach relevant *µ* values (Table 7).

Although not studied in this study by direct measurements, the porosity is one of the most important characteristics of mortars, particularly influencing their behavior in terms of strength, permeability, water absorption, and one of the main properties considering compatibility requirements. For this reason, the influence of the mortars’ porosity on their final physico-mechanical properties will be one of the subjects of the future works.

## 4. Conclusions

The effect of different binder/aggregate ratios and air lime additions on the final physical-mechanical properties prepared from commercially available Natural cements Prompt Vicat (France), Marfil (Spain), and Tigre (Spain) was investigated. The broad experimental campaign comprised assessment of chemical and mineralogical characteristics of three Natural cements; and mineralogical, morphological, mechanical, and physical properties of the mortars, such as water absorption by capillarity, water vapor permeability, and water vapor diffusion resistance. The following results and findings can be highlighted:

- Natural cements Prompt Vicat (NCPV), Marfil (NCM) and Tigre (NCT) show very similar chemical and mineralogical composition.
- Mortars prepared from Natural cements show comparable mineralogical composition, however resulting in the mortars with distinct mechanical, water adsorption, and water vapor permeability properties.
- No gradual changes in mechanical strengths and water capillarity values are observed with curing time and majority of the mortars show comparable values at 28, 60, and 90 days.
- The mortars poorer in aggregate (1:0.5 > 1.1 > 1.5 binder/aggregate) and poorer in air lime in mortar’s formulation (0.75:0.25 > 0.5:0.5 NC:lime) have in general better physical-mechanical properties.
- To reach compatibility requirements with original materials, each NC requires a specific mortar formulation, therefore Natural cements variability needs to be taken into consideration, when selecting a proper binder for the conservation and rehabilitation of historic renders and plasters.
- Mortars developed in this study show compatibility in terms of physical-mechanical properties with several original historic mortars from late 19th and the beginning of 20th century with the values of compressive strength (8–14.5 MPa), water absorption by capillarity (<20 kg·m^−2^·h^−0.5^), water vapor permeability (<4 × 10^−10^ kg·s^−1^·m^−1^·Pa^−1^), and water vapor diffusion resistance (<28).

The results of this study provide a unique and important data collection that may play an important role in choosing an adequate mortar for a specific restoration application (e.g., renders).

## Figures and Tables

**Table 1:** Mix proportions and water requirements.

ID	Binder	Reference	Mix Proportions (Volumetric Ratio) (b/a)	Water (wt.%)
1	Natural Hydraulic Lime NHL 3.5	1_NHL	1:2	15.33
2	Natural Hydraulic Lime 3.5 + Air Lime CL90	2_NHL:CL90	0.5:0.5:2	17.00
3	Natural Cement Prompt Vicat	3_NCPV	1:0.5	21.21
4	Natural Cement Prompt Vicat	4_NCPV	1:1	17.67
5	Natural Cement Prompt Vicat	5_NCPV	1:2	15.00
6	Natural Cement Prompt Vicat + Air Lime CL90	6_NCPV:CL90	0.75:0.25:1	16.67
7	Natural Cement Prompt Vicat + Air Lime CL90	7_NCPV:CL90	0.5:0.5:1	16.67
8	Natural Cement Prompt Vicat + Air Lime CL90	8_NCPV:CL90	0.75:0.25:1.5	15.33
9	Natural Cement Prompt Vicat + Air Lime CL90	9_NCPV:CL90	0.5:0.5:1.5	16.33
10	Natural Cement Marfil	10_NCM	1:0.5	35.67
11	Natural Cement Marfil	11_NCM	1:1	20.00
12	Natural Cement Marfil	12_NCM	1:2	22.67
13	Natural Cement Marfil + Air Lime CL90	13_NCM:CL90	0.75:0.25:1	17.50
14	Natural Cement Marfil + Air Lime CL90	14_NCM:CL90	0.5:0.5:1	25.00
15	Natural Cement Marfil + Air Lime CL90	15_NCM:CL90	0.75:0.25:1.5	19.67
16	Natural Cement Marfil + Air Lime CL90	16_NCM:CL90	0.5:0.5:1.5	17.33
17	Natural Cement Tigre	17_NCT	1:0.5	24.17
18	Natural Cement Tigre	18_NCT	1:1	20.00
19	Natural Cement Tigre	19_NCT	1:2	18.00
20	Natural Cement Tigre + Air Lime CL90	20_NCT:CL90	0.75:0.25:1	20.17
21	Natural Cement Tigre + Air Lime CL90	21_NCT:CL90	0.5:0.5:1	19.33
22	Natural Cement Tigre + Air Lime CL90	22_NCT:CL90	0.75:0.25:1.5	16.67
23	Natural Cement Tigre + Air Lime CL90	23_NCT:CL90	0.5:0.5:1.5	17.00

**Table 2:** Specific gravity of materials.

Material	Specific Gravity (kg/m^3^)
Air Lime CL90	385 ± 3
NHL 3.5	744 ± 5
Natural Cement Prompt Vicat NCPV	791 ± 5
Natural Cement Marfil NCM	847 ± 6
Natural Cement Tigre NCT	911 ± 6
Siliceous River Sand	1559 ± 7

**Table 3:** Chemical analysis of materials (wt.%).

Material	NHL 3.5	CL90	NCPV	NCM	NCT	Sand
Na_2_O	0.06	nd **	0.16	0.15	0.32	nd
MgO	0.58	0.16	3.11	1.46	5.99	nd
Al_2_O_3_	1.66	nd	5.93	7.83	5.26	2.01
SiO_2_	12.28	nd	13.44	18.96	16.32	96.45
P_2_O_5_	0.04	0.01	0.05	0.06	0.07	0.02
SO_3_	0.45	nd	4.12	3.50	2.16	nd
Cl^−^	0.01	nd	0.01	0.01	0.02	nd
K_2_O	0.16	0.01	1.25	1.50	1.39	1.14
CaO	65.61	62.24	50.47	52.18	51.61	0.09
TiO_2_	0.16	0.03	0.24	0.37	0.25	0.09
MnO	0.01	0.01	0.06	0.07	0.05	0.01
Fe_2_O_3_	0.49	0.09	2.85	2.97	2.32	0.20
LOI *	18.46	38.12	18.05	10.77	13.64	0.22

* Loss on ignition. ** nd—not detected.

**Table 4:** Mineralogical composition of materials identified by XRD analysis.

Material	Chemical Formula	Mineral Phase
Siliceous River Sand	SiO_2_	Quartz
	KAlSi_3_O_8_	Microcline
Natural Hydraulic Lime **NHL 3.5**	Ca(OH)_2_	Portlandite
	Ca(CO_3_)	Calcite
	Ca_2_SiO_4_	Belite (*β*-C_2_S)
	SiO_2_	Quartz
Air Lime **CL90**	Ca(OH)_2_	Portlandite
	Ca(CO_3_)	Calcite
Natural Cement Prompt Vicat **NCPV** Natural Cement Marfil **NCM** Natural Cement Tigre **NCT**	Ca(CO_3_)	Calcite
	CaMg(CO_3_)_2_	Dolomite
	Ca_2_SiO_4_	Belite (*α*′-C_2_S)
	Ca_2_SiO_4_	Belite (*β*-C_2_S)
	Ca_2_Al[AlSiO_7_]	Gehlenite (C_2_AS)
	Ca_5_Si_2_O_7_(CO_3_)_2_	Tilleyite
	Ca_5_(SiO_4_)_2_(CO_3_)	Spurrite
	4CaO·Al_2_O_3_·Fe_2_O_3_	Brownmillerite (C_4_AF)
	SiO_2_	Quartz

**Table 5:** Mineralogical composition of mortars identified by XRD analysis.

Mortar	Chemical Formula	Mineral Phase
Natural Hydraulic Lime **1_NHL 3.5**	Ca(OH)_2_	Portlandite
	Ca(CO_3_)	Calcite
	Ca_2_SiO_4_	Belite (*β*-C_2_S)
	Ca_3_Si_2_O_7_*·*3H_2_O	Calcium Silicate Hydrate (C–S–H)
	SiO_2_	Quartz
Natural Cement Prompt Vicat **3_NCPV** Natural Cement Marfil **10_NCM** Natural Cement Tigre **17_NCT**	Ca(CO_3_)	Calcite
	CaMg(CO_3_)_2_	Dolomite
	Ca_2_SiO_4_	Belite (*α*′-C_2_S)
	Ca_2_SiO_4_	Belite (*β*-C_2_S)
	Ca_2_Al[AlSiO_7_]	Gehlenite (C_2_AS)
	Ca_5_Si_2_O_7_(CO_3_)_2_	Tilleyite
	Ca_5_(SiO_4_)_2_(CO_3_)	Spurrite
	4CaO·Al_2_O_3_·Fe_2_O_3_	Brownmillerite (C_4_AF)
	(CaO)_6_(Al_2_O_3_)(SO_3_)_3_·32H_2_O	Ettringite
	SiO_2_	Quartz

**Table 6:** Carbonation of mortars after 28, 60, and 90 days.

		Carbonation (mm)		
Reference	Mix Proportions	28 d	60 d	90 d
1_NHL	1:2	2.10 ± 0.06	6.90 ± 0.13	7.5 ± 0.09
2_NHL:CL90	0.5:0.5:2	3.50 ± 0.08	7.30 ± 0.14	8.5 ± 0.15
3_NCPV	1:0.5	4.00 ± 0.08	Total	Total
4_NCPV	1:1	4.50 ± 0.08	Total	Total
5_NCPV	1:2	10.50 ± 0.10	Total	Total
6_NCPV:CL90	0.75:0.25:1	2.00 ± 0.06	2.80 ± 0.07	4.80 ± 0.11
7_NCPV:CL90	0.5:0.5:1	2.20 ± 0.06	3.40 ± 0.09	5.50 ± 0.11
8_NCPV:CL90	0.75:0.25:1.5	3.50 ± 0.08	5.50 ± 0.12	6.50 ± 0.10
9_NCPV:CL90	0.5:0.5:1.5	4.00 ± 0.09	6.20 ± 0.12	7.00 ± 0.12
10_NCM	1:0.5	Total	Total	Total
11_NCM	1:1	Total	Total	Total
12_NCM	1:2	Total	Total	Total
13_NCM:CL90	0.75:0.25:1	2.20 ± 0.06	4.60 ± 0.10	5.00 ± 0.09
14_NCM:CL90	0.5:0.5:1	1.60 ± 0.06	5.10 ± 0.12	5.50 ± 0.10
15_NCM:CL90	0.75:0.25:1.5	3.20 ± 0.10	5.90 ± 0.12	8.40 ± 0.18
16_NCM:CL90	0.5:0.5:1.5	3.90 ± 0.10	9.50 ± 0.18	9.60 ± 0.20
17_NCT	1:0.5	2.00 ± 0.06	2.60 ± 0.07	4.60 ± 0.07
18_NCT	1:1	2.60 ± 0.08	4.70 ± 0.09	6.30 ± 0.09
19_NCT	1:2	6.10 ± 0.12	10.00 ± 0.20	13.90 ± 0.25
20_NCT:CL90	0.75:0.25:1	2.00 ± 0.07	4.00 ± 0.10	5.10 ± 0.08
21_NCT:CL90	0.5:0.5:1	2.90 ± 0.07	5.10 ± 0.10	6.20 ± 0.09
22_NCT:CL90	0.75:0.25:1.5	3.60 ± 0.10	6.10 ± 0.11	8.40 ± 0.10
23_NCT:CL90	0.5:0.5:1.5	3.90 ± 0.10	8.10 ± 0.10	9.70 ± 0.12

**Table 7:** Water vapor permeability and resistance coefficient of mortars at 28 days of curing.

Reference	Mix Proportions	Water Vapor Permeability (10^−11^)(kg·s^−1^·m^−1^·Pa^−1^)	Water Vapor Diffusion Resistance Coefficient (*µ*)
1_NHL	1:2	1.64	11.85
2_NHL:CL90	0.5:0.5:2	1.61	12.01
3_NCPV	1:0.5	1.37	14.12
4_NCPV	1:1	1.33	14.57
5_NCPV	1:2	1.27	15.32
6_NCPV:CL90	0.75:0.25:1	1.30	14.89
7_NCPV:CL90	0.5:0.5:1	1.24	15.67
8_NCPV:CL90	0.75:0.25:1.5	1.25	15.47
9_NCPV:CL90	0.5:0.5:1.5	1.26	15.41
10_NCM	1:0.5	1.35	14.36
11_NCM	1:1	1.33	14.61
12_NCM	1:2	1.32	14.66
13_NCM:CL90	0.75:0.25:1	1.26	15.36
14_NCM:CL90	0.5:0.5:1	1.23	16.73
15_NCM:CL90	0.75:0.25:1.5	1.35	15.14
16_NCM:CL90	0.5:0.5:1.5	1.28	16.41
17_NCT	1:0.5	0.70	27.78
18_NCT	1:1	0.69	28.10
19_NCT	1:2	0.60	32.24
20_NCT:CL90	0.75:0.25:1	0.68	28.46
21_NCT:CL90	0.5:0.5:1	0.66	29.55
22_NCT:CL90	0.75:0.25:1.5	0.66	29.34
23_NCT:CL90	0.5:0.5:1.5	0.64	30.11

## Data Availability

Not applicable.
